# Using the ADDIE model to design and develop physical education lessons incorporated with a functional training component

**DOI:** 10.3389/fpubh.2023.1201228

**Published:** 2023-09-22

**Authors:** Hailing Li, Jadeera Phaik Geok Cheong

**Affiliations:** ^1^Faculty of Sports and Exercise Science, Universiti Malaya, Kuala Lumpur, Malaysia; ^2^Department of Physical Education, Putian University, Putian, Fujian, China; ^3^UM STEM, Universiti Malaya, Kuala Lumpur, Malaysia

**Keywords:** instructional design, functional training program, primary school, school children, physical fitness

## Abstract

**Introduction:**

Good physical fitness is the foundation of a healthy lifestyle. For students, the school becomes the main place to improve their physical fitness. The traditional physical education class places little emphasis on improving physical fitness and students’ physical fitness have continued to decline. To address these challenges, this study aimed to design and develop a functional training program that can be incorporated into existing physical education lessons to improve students’ physical fitness levels.

**Methods:**

This study adopted the instructional design framework of the Analysis, Design, Development, Implementation, Evaluation (ADDIE) model to guide the design and development of the functional training program. After development, the program was implemented and evaluated.

**Results:**

The results showed the program aroused students’ interest and significantly improved students’ physical fitness.

**Conclusion:**

This study showed the usefulness of functional training in improving the physical fitness of primary school students. In addition, it provided a reference for how to use functional training in conjunction with the physical education syllabus.

## 1. Introduction

Good physical fitness is a necessary condition for an effective life, not only to help individuals work efficiently such as when doing housework or driving a car but also to enjoy leisure time or deal with emergencies (1, 2). For children, one of the ways to improve physical fitness was through the school system. It was previously reported that physical education (PE) in schools was considered the ideal intervention point to improve the current and future population health by increasing children’s physical fitness levels (3, 4). However, the leader of the Chinese national primary and secondary school PE and health curriculum standards development group had argued that in China, PE had evolved into a “safety class,” “discipline class,” and “military class” that emphasized consistency in learning and practicing physical skills and keeping students in a specific position for long periods, which results in children not even sweating at the end of a class (5). Such PE benefits neither public health nor the students’ physique (6). Faced with the problems of the traditional PE syllabus, there is an urgent need to conduct an in-depth study of PE to find solutions to improve the quality of the PE syllabus and effectively increase students’ physical fitness. Ji (7) proposed a healthy physical education curriculum model (HPECM), which stipulated that each class should include 20 min of skill exercise and 10 min of physical fitness exercise. The 10 min of physical fitness exercises were primarily intended to address the traditional PE lesson which did not include any time dedicated to physical fitness (6). Besides the allocation of time, the program should comprise physical fitness exercises which were diversified, interesting, and act as compensatory methods and means to deal with the traditional PE lesson of learning only one motor skill and developing only a specific body part in each lesson.

One way to improve physical fitness is through functional training. Functional training began in sports medicine, then was introduced to the field of athletic training by coaches, subsequently being introduced to gyms, where the general public had access to a vast array of methods associated with it (8). Nowadays, functional training has become a fitness hotspot. Since 2007, according to the global fitness trend surveys conducted by the American Society of Sports Medicine (ACSM), functional training has always been listed among the Top 20 fitness regimes (9–23). Most importantly, functional training was found to provide health benefits and could be used to improve the ability to perform a variety of activities of daily living at home, at work, or during play, without the risk of excessive injury or fatigue (24). Besides health benefits, functional training could contribute to performance benefits in the areas of speed, strength, and power as pointed out by Boyle (25). Finally, the advantages of needing little space, equipment, and time also contribute to its popularity (26). Given the superiorities of functional training, some researchers tried to transplant functional training to school PE. For example, Oliver and Brezzo (27) examined the effects of functional balance training on female collegiate and found that functional balance training could strengthen the core while simultaneously balancing activities, which not only aided functional performance but also helped prevent injury. Likewise, Lasković et al. (28) compared the effects of functional training and PE classes on the physical fitness of primary school girls and found that both training methods had a positive impact on the physical fitness of the students. Despite these efforts, the literature on the use of functional training in PE curricula is limited, particularly in primary schools.

This study was conducted to design and develop a 12-week functional training program that could be incorporated into existing PE lessons. The design and development were based on the ADDIE model. The study could verify the feasibility of incorporating functional training into the primary school PE curriculum as well as provide a reference for other researchers who were interested in improving the effectiveness of PE.

## 2. Pedagogical framework

The study mainly used the ADDIE framework to guide the process of designing and developing a functional training program. The ADDIE framework is a process model developed for the military and enthusiastically adopted by the army due to its clear delineation of instructional design steps, namely Analysis, Design, Development, Implementation, and Evaluation (29, 30). The generic and dynamic process within five stages allowed instructional designers to use the model with flexibility, in terms of how different steps or procedures were performed, which has made it one of the most popular and frequently cited instructional design models (29, 31) and applied in various disciplines (32). In education, it has been referred to in the subject of English language (33–35), chemistry (36) and physics (37), as well as in a nursing curriculum (38). Additionally, some studies had applied the ADDIE model to the PE curriculum. For example, Wei (39) shared how the “micro-lecture” designed and produced based on the ADDIE model was applied to the teaching of PE in schools and was favored by the teachers and students. Yan (40) also discussed how to improve the level of social sports instructors by using the ADDIE model to design training courses.

## 3. Learning environment

### 3.1. Physical education curriculum setting

PE in primary school is the basic organizational form of PE teaching and an important part of the school curriculum in China. It plays an important role in improving students’ physical health, enhancing social adaptability, and cultivating students’ awareness of lifelong PE (41). During the development of this program, it was necessary to consider the characteristics of the PE curriculum setting in the primary schools of China, which were as follows.

First, each PE lesson had a total of 40 min. Second, the lesson had four parts, which were the initial part, the preparatory part, the fundamental part, and the final part. Third, the teaching tasks of different parts were also different. The initial part focused on organizing students, focusing on students’ attention, and preparing for class. The preparatory part, also known as the warm-up part, was to improve muscle temperature and prevent sports injuries, improve the function of internal organs, and adjust mental state (42). The fundamental part was the key link of PE, and students learned and mastered new knowledge in this part. The fundamental part included two parts when arranging the teaching content, one was the main teaching material part, and the other was the auxiliary teaching material part (43). The main teaching material focused on learning motor skills or sports tactics, while the auxiliary material was generally on games, competitions, etc. as a supplement and continuation of the main material (44). The main teaching material must be arranged in strict accordance with the contents of the skill materials prescribed by the Ministry of Education, while the auxiliary teaching materials could be arranged freely according to the actual situation of the school, weather conditions, teachers’ specialties, and so on. The final part was to reduce the excitability of the cerebral cortex, relax, and relieve muscle fatigue.

### 3.2. Primary school students’ growth characteristics

Understanding and mastering the characteristics of children’s growth, such as the skeletal system, cardiovascular system, respiratory system, and nervous system, was critical for guiding the development of the PE curriculum. In terms of the skeletal system of students, their bones were elastic but had poor hardness and firmness and were easy to bend and deform; their range of joint motion was more extensive than that of adults, but the firmness was relatively poor; their muscles were weak contractile function, poor endurance, and easy fatigue (45–48). In the area of cardiovascular system, school children’s heart development was not yet complete, whereby myocardial fibers were thin, and contractility was weak (45–48). The respiratory system was characterized by the ability to adapt to exercises with higher intensity and longer duration was lower and fatigue quickly (45–48). In terms of the nervous system, nerve cells in childhood had poor working endurance and were prone to fatigue (45, 46, 48).

### 3.3. Functional training modules

Athletes’ Performance Institute (API), the functional training center established in 1999, had been widely acclaimed for its advanced training system, which classified functional training as seven modules: pillar preparation, movement preparation, plyometric training, movement skill training, strength training, energy systems development, and recovery and regeneration (49) (Table 1). The innovative training system attracted the attention of Chinese sports experts. In September 2011, the General Administration of Sport of China cooperated with API and introduced functional training to China (50). The training system built by API became one of the most important reference systems for the application of functional training in China and is also the reference system for this study.

**Table 1:** List of functional training modules.

Module	Description
Pillar preparation	This module solves the limitations of individual joint flexibility or stability, corrects the compensated movement pattern, reduces the energy leakage, strengthens the hips, torso, and shoulder strength of the spine, and coordinates the muscle power sequence, to prepare the athletes for posture adjustment and exercise load.
Movement preparation	This module increases the core temperature of the human body, activates the greater body inertia gluteus maximus, strengthens the nervous system excitement, integrates the movement pattern, and increases muscle elasticity. to improve the overall efficiency of training.
Plyometrics	This module makes use of the characteristics of muscle and tendon to store elastic potential energy and stretch reflex to improve the collective acceleration and deceleration ability and explosive force and to improve the energy transfer efficiency between joints.
Movement skills	This module according to the special characteristics and training objectives, through the linear speed, lateral speed, and multi-speed exercises, effectively improve the response speed, sensitivity, mobility, and explosive force, and reduce the risk of injury.
Strength and power	Through multi-joint and multi-movement plane movement strength training, this module can improve stability strength, optimize movement patterns, integrate the energy transfer of movement chain, and reduce the risk of sports injury.
Energy system development	This module specifically develops the cardiovascular system and energy metabolism system according to the heart rate interval corresponding to exercise intensity.
Regeneration and recovery	This module can help restore the initial length of muscles, accelerate the repair of muscle fibers, and stimulate blood and lymph circulation through the stretching and soft tissue relaxation of muscles, ligaments, and fascia, thus relieving fatigue and speeding up the recovery process.

## 4. Learning objectives

The objectives of PE for primary school students were that through the study of PE and health curriculums, students would master various physical training methods, and could actively participate in various physical exercises, and their physical fitness levels would be significantly improved, meeting the corresponding requirements of the 2014 revised Chinese National Student Physical Fitness Standard (CNSPFS) (51).

## 5. Pedagogical format–functional training module design using ADDIE

### 5.1. Analysis

During the analysis stage, a needs analysis was mainly carried out to differentiate between their current level of physical fitness and what they needed to improve by the end of the lessons (52). Firstly, the 2014 revised CNSPFS battery (53) was carried out to assess the students’ physical fitness to understand their current physical condition (54). The CNSPFS test consisted of Body Mass Index (BMI) for body composition, vital capacity (VC) for lung function, 50 m sprint for speed, sit and reach for flexibility, timed rope-skipping for coordination and limbs strength, timed sit-ups for abdominal strength, and 50 m × 8 shuttle run for aerobic capacity (55). Details of each test and the procedures for data collection have been detailed by Li and colleague (54). Secondly, to guide the program design and development and confirm items for consideration, five experts were interviewed online, including three functional training experts and PE experts with more than 18 years of teaching experience.

### 5.2. Design

At this stage, a 12-week program was proposed to develop students’ physical fitness based on the feedback from the first stage. The program was divided into three stages: basic stage, advantage stage I, and advantage stage II. The previous stage was the foundation of the following stage. It was arranged in the auxiliary part of the lesson according to the characteristics of the PE curriculum setting and HPECM.

The first step was the choice of content. According to the results of the analysis stage, the speed of students’ physical fitness dropped sharply (54). Therefore, when choosing the functional training component, on the premise of achieving the overall physical fitness goal, we focused on incorporating the development of speed into the 12-week PE lesson, such as movement skills, which helped to improve speed level. Next was the arrangement of the contents. Due to prepubescent children having immature skeletons, children in primary school should not participate in excessive amounts of vigorous-intensity exercise (56). During this period, the program arranged for students to develop physical fitness based on the mastery of basic movement patterns. On the other hand, students’ bones were relatively soft, with more cartilage and the supporting force of joints is weak. Therefore, the physical fitness exercises should avoid using some heavy weight, heavy load equipment in the program which would hinder the development of students’ bones and result in spine deformation. So, the advanced stages combined the medicine ball, agility ladder, and cone to develop the physical fitness of the students. Then, when arranging the workload, overcoming the student’s own weight or applying a light load was generally used. Changes in load were achieved in the form of (1) variations in training routes from unidirectional to multidirectional, and (2) distances and the number of repetitions.

### 5.3. Development

This phase was to develop the program and mainly included three steps. Firstly, according to the knowledge obtained in the first two stages of the model, the corresponding functional training components were selected and incorporated into the 12-week PE syllabus. According to the school’s PE timetable and schedule, there were three PE lessons a week, contributing to a total of 36 functional training programs that were developed. All the movements within the program were recorded into videos to assist teaching and learning. Secondly, when completing the development of the 12-week program, to validate the draft program and ensure a variety of voices were heard, the developed materials were sent to the same experts as those from the analysis phase and to six additional PE teachers with at least 6 years of teaching experience for their review of the proposed program. The data were collected by WeChat which is a widely popular social media in China (57–59) and analyzed by NVivo 12. The final step was to modify and form the final program.

### 5.4. Implementation

The implementation was the process of transforming a plan into action (60). In this implementation stage, the proposed 12-week functional training program was incorporated into 36 PE lessons. Since primary school students are categorized into three levels of learning, with grades 1–2 being Level One, grades 3–4 being Level Two, and grades 5–6 being Level Three in China (61), therefore, six students in good health and developing normally were randomly sampled at each level of a primary school in Beijing, respectively, and the final combination of 18 students (M age = 9.67 ± 1.75 years) was used as participants in the study. Before implementation, this study was approved by the University Malaya Research Ethics Committee (Approval number: UM.TNC2/UMREC – 667) and the participating school and all parents/guardians of participating students signed the consent form.

### 5.5. Evaluation

**Figure 1:**
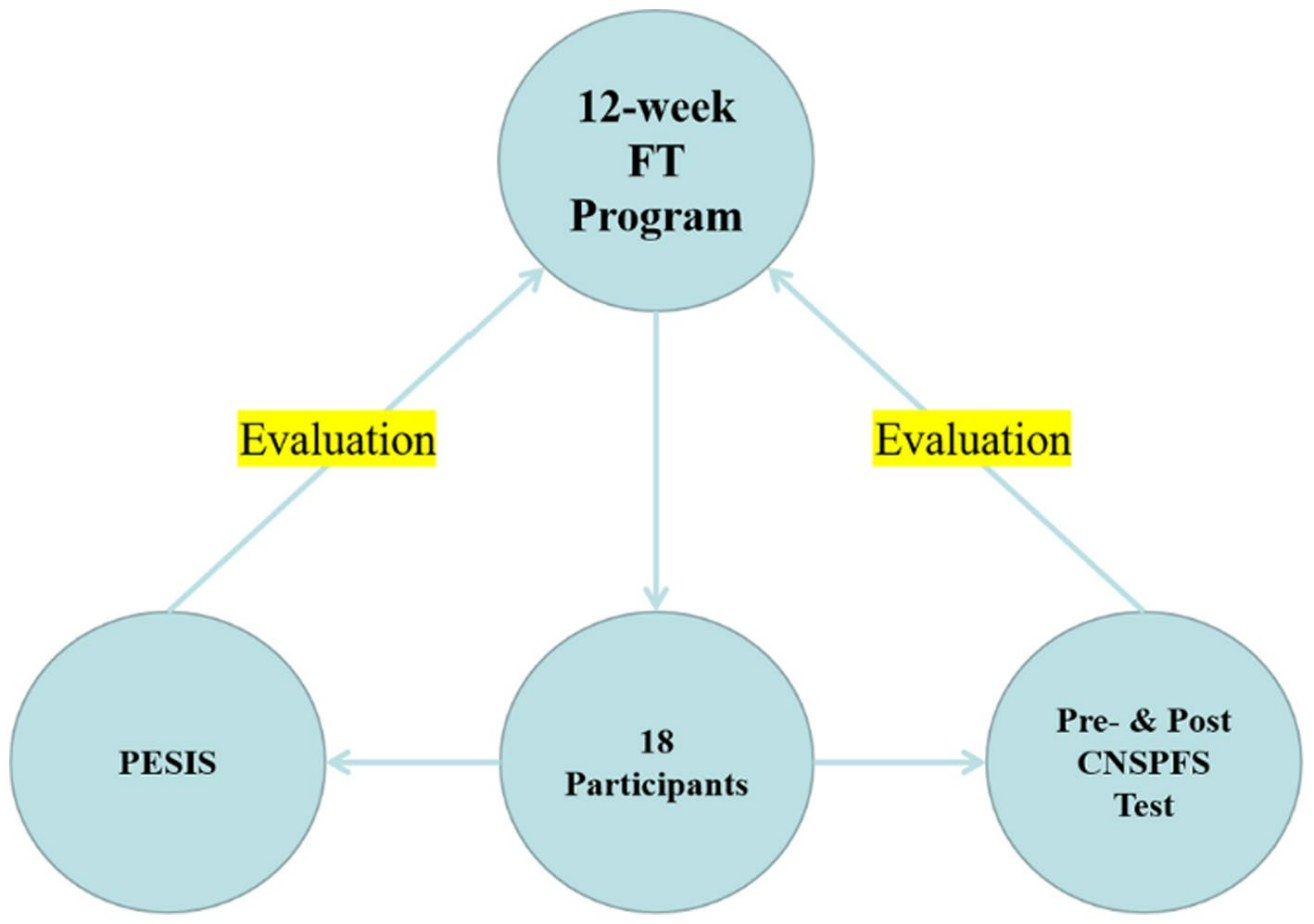
Flowchart of the evaluation.

The last phase of the instructional design process was evaluation, which helped identify the real value of the program (60, 62). This stage evaluated two items: (i) situational interest and (ii) physical fitness (Figure 1). Students’ situational interests were measured to determine whether the program could stimulate students’ motivation to learn. Situational interest, defined as the attractiveness of an activity or learning task to an individual (63), was usually triggered by external stimuli (64) and had been regarded as a motivating factor to be particularly useful to educators (65). According to the constructivism learning theory, learner motivation was an important factor in learning (64, 66). Situational interest, as one of the sources of motivation, could effectively motivate learners to actively participate in the learning process (65), especially for learners in the adaptation or naive stage, where situational interest was the only motivation that attracted learners to focus on the learning process (65, 67). The measurement tool used was the Chinese version of the PE Situational Interest Scale (PESIS), which is suitable for elementary school students (Cronbach *α*: 0.81–0.94) (68). The Chinese version of PESIS is composed of 24 short sentences, with a total of six dimensions, namely Instant Enjoyment (IE), Novelty (N), Challenge (C), Attention Demand (AD), Exploration Intention (EI), and Total Interest (TI), each dimension has four questions, and the distribution of each question in the scale is random. The scale uses a Likert five-point scale with five options, namely “strongly agree,” “agree,” “neutral,” “disagree,” and “strongly disagree,” representing the scores are 5, 4, 3, 2, 1, respectively. The Statistical Package for the Social Science (SPSS) software (version 25.0) for Windows was used to analyze the data.

As for physical fitness, the 2014 revised CNSPFS test battery was used to measure the physical fitness of the students before and after the intervention. Baseline data were obtained from the analysis phase, while the post-test was conducted after the implementation phase, at the end of the 12-week functional training program.

## 6. Results

According to the 2014 revised CNSPFS test conducted on the students, the current status of students’ fitness showed that the 50 m sprint dropped sharply which needed to be improved (54). Combined with the results of the thematic analysis of the five expert interviews using NVivo software (Table 2), the final 36 functional training programs were drafted and developed, which included three stages: 2 weeks of basic stage mainly used to learn the base movement pattern; 5 weeks of Advance stage I; and 5 weeks of Advance stage II. Video recordings of the corresponding movements of the functional training program were made to assist students in learning so that students had an intuitive understanding of movements. The summary functional training program that was drafted and developed can be found in Table 3. The complete program for each of the 36 sessions can be found in an experimental study, which also includes the arrangement of the control group (69).

**Table 2:** Results of the subject matter experts interviews.

Theme	Description
Content arrangement	*The content should choose some more interesting exercise movements.*
	*The content should be simple and easy for students to practice.*
	*The content should consider the sensitive period of students’ physical development.*
Workload arrangement	*The arrangement of load should be arranged progressively.*
	*The load needs to be arranged according to the student’s actual situation.*
	*The load should be arranged flexibly and can be adjusted in practice.*
Training equipment	*The equipment should be selected to be safe and easy to operate.*
	*The equipment should be selected according to the size of the playground.*
	*It is best to choose self-body weight training without equipment.*
Order arrangement	*Skill content should be arranged before physical fitness exercises content. If the intensity of the skill content is relatively high, physical fitness exercises that require a high level of neural excitement can be prioritized over the skill content.*
	*If the intensity of the skill content is relatively high, physical fitness exercises that require a high level of neural excitement can be prioritized over the skill content.*
	*If the movement is not proficient, the students will not train very well. Therefore, the movements should be extended based on the mastery of the movements.*

**Table 3:** The draft functional training program.

Stage	Module	Session	Exercise selection
Basic stage	Movement pattern	1	Squats; lateral squats; jump squats; squat turn 90°
		2	Squats; lateral squats; jump squats; squat turn 90°
		3	Jump squats; bounds; squat turn 180°
		4	Forward lunge; lateral lunge; backward lunge
		5	Walking lunges; walking lunges + rotation; backward walking lunge; backward walking lunges+ rotation
		6	Seated step; stride; carioca; trot; bound; sprint
Advantage stage I	Plyometric training (line drill)	7	Hop back and forth; lateral hop; one leg hop back and forth; one leg lateral hop; jack jump; cross jump
		8	Forward jumps; one leg forward jump; skipping; “Z” bounds
		9	forward jumps + sprint; one leg forward jump + sprint; skipping + sprint; “Z” bounds + sprint
	strength and power (medicine ball)	10	Squats with a ball; lateral squats with a ball; squats with a ball pushing forward; squats with a ball pushing up; lateral squats with a ball pushing forward; lateral squats with a ball pushing up
		11	Forward; lateral, and backward squats with a ball; forward, lateral, and backward squats with a ball pushing forward; forward, lateral, and backward squats with a ball pushing up
		12	Diagonal chop down; diagonal chop up; deadlift with a ball; squat jumping with a ball; bound with a ball; squat jumping turn 90° with a ball
	Strength and Power (pad)	13	Plank; supination-abdominal crunch; plank-hand touch shoulder; supination-reverse crunch
		14	“V” sit with rotation; Supination-leg rotation; pronation-hyperextension; supination- 45-degree abdominal crunch; supination-leg raise; hyperextension
		15	Glute bridge; supination-arm overhand sit with straight legs; supination-leg raise; glute bridge - one leg lift; V-up
	Movement skill (agility ladder)	16	One foot in each rung; two feet in each rung; high knees; forward carioca; in-in-out-out
		17	One foot in each rung; two feet in each rung; high knees; forward carioca; in-in-out-out
		18	One foot in each rung + sprint; two feet in each rung + sprint; high knees + sprint; forward carioca + sprint; In-in-out-out + sprint
	Energy systems development (cone)	19	5–10 shuttles verbal commands
		20	5–10-15 shuttles
		21	5–10–15-20 shuttles
Advantage stage II	Plyometric training (line drill)	22	Hops; “Z” hops; hop scotch two feet in; hop scotch one foot in
		23	One leg hop; one leg “z” hop
		24	Lateral hop; forward and backward Lateral hop; one leg lateral hop; one leg forward and backward hop
	Strength and Power (medicine ball)	25	squats with a ball; lateral squats with a ball; squats with a ball pushing Forward; squats with a ball pushing up; lateral squats with a ball pushing forward; lateral squats with a ball pushing up
		26	Walking lunges pushing forward; walking lunges pushing up; walking lunges with a rotation
		27	Walking lunges pushing forward; walking lunges pushing up; walking lunges with a rotation
	Strength and Power (pad)	28	Plank; supination- abdominal crunch; plank – hand touch shoulder; supination-reverse crunch
		29	“V” sit with rotation; supination-leg rotation; pronation-hyperextension; supination- 45-degree abdominal crunch; supination-leg raise; hyperextension
		30	Glute bridge; supination-arm overhand sit with straight legs; supination-leg raise; glute bridge - one leg lift; V-up
	Movement skill (agility ladder)	31	Sprint (two cones); side slide (two cones); shuttles; “S” running; running circle; lateral running circle
		32	“L” sprint; “L” sprint + side slide; “L” sprint + carioca running; “V” sprint; “V” sprint + side slide; “V” + “8” circle running
		33	Square sprint; square side slide + sprint; square carioca + sprint + side slide; square running circle; “X” sprint; agility box (verbal commands)
	Energy systems development (cone)	34	5–10-15 shuttles
		35	5–10–15-20 shuttles
		36	5–10–15-20-25 shuttles

### 6.1. Expert evaluation

After reviewing the draft program, the experts gave their feedback, which was analyzed by NVivo 12 from word cloud and Sentiment.

**Figure 2:**
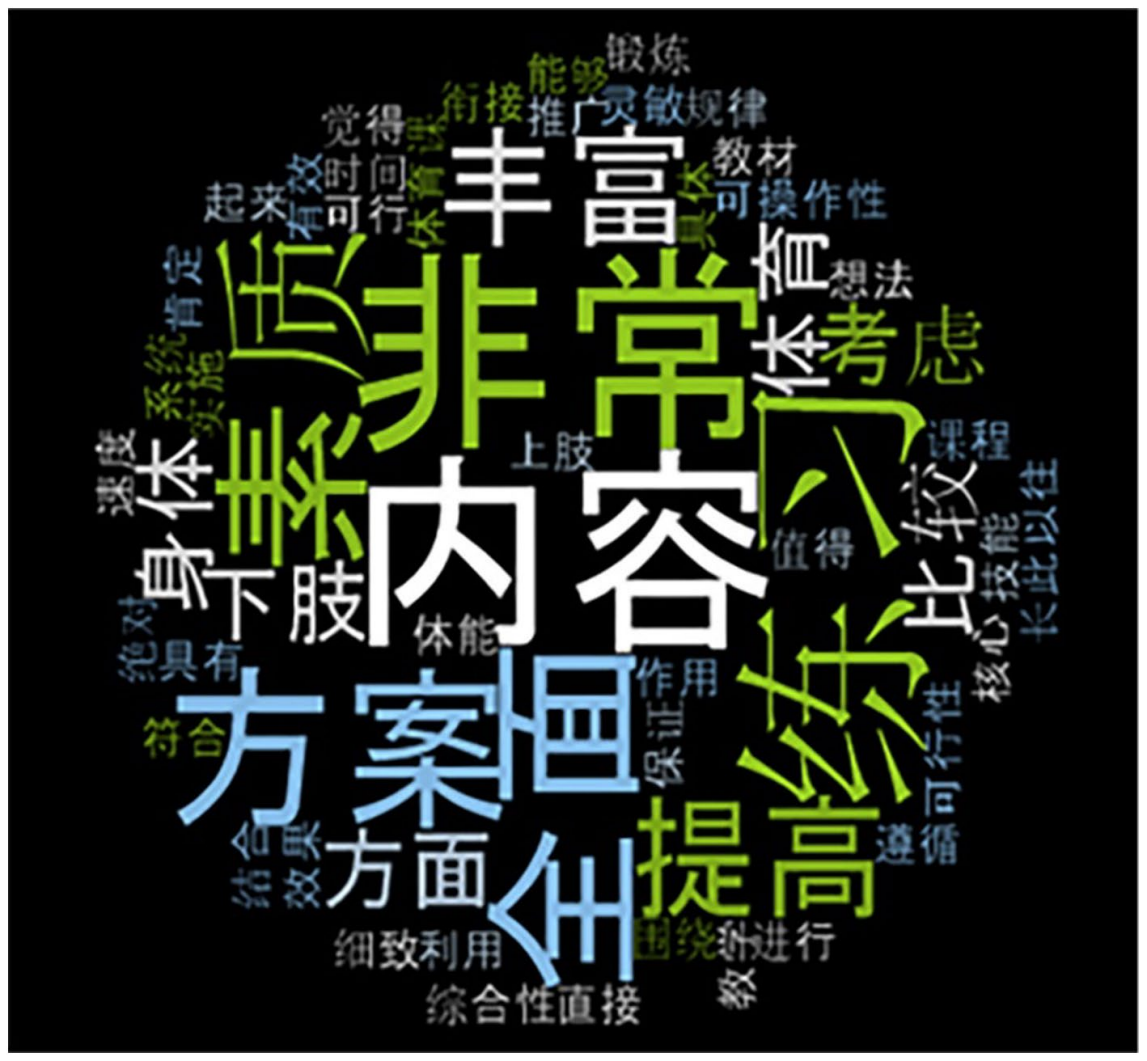
Word cloud generated from expert feedback.

Words commonly used to express positive or negative feelings are the most important indicators of feelings (70). The data results generated by the word cloud function of NVivo analysis software show the most common words used by experts in the macro evaluation of the program. The most common words found in the evaluations, according to the results of NVivo were: ‘全面’ (comprehensive) and ‘丰富’ (rich), followed by ‘细致’ (meticulous), ‘综合性’ (synthesis), ‘可操作性’ (Operability), and ‘可行性’ (feasibility; Figure 2).

Sentiment identification also called subjectivity identification is to determine whether the opinion orientation in the given text is position, negative, or neutral (71). The sentiment analysis results of the overall evaluation are that the majority of interviewees held a positive attitude toward it, accounting for 90.91%, while 9.09% of the respondents had moderately negative emotions, and thought that this time-length content arrangement would face some extraneous variables, such as class discipline, cognitive level, class size, and teaching tasks during the implementation process. In response to these extraneous variables, this program had taken targeted measures to address them. First of all, poor student discipline in a classroom would disrupt the flow of lessons and conflict with both learning objectives and processes, as the attention of teacher and students shifted away from the teaching contents at hand to the distractions provided by disruptive behaviors (72). Therefore, in facing the extraneous variables, participating teachers should be trained to create stronger bonds among the students, and to establish an interactive educational environment to cope with students’ disruptive behaviors (73). Further training was needed on how to integrate the content of textbook materials and proposed program content, and how to organize students to ensure that the proposed program would be completed. When faced with the lower cognitive abilities of primary students, the observational learning strategy is recommended, which was an effective method of motor skills learning (74). During the lesson of the program, in the theory class of the term, the teacher used multimedia to show the recorded experiment movement videos in advance. Through multi-angle display, slow motion playback, zooming in on detailed shots, etc., video actions are demonstrated to students frame by frame, which improved the standard and intuitiveness of the presentation, and ensured that students could clearly understand each key action and detail and could carry out correct imitation and learning.

### 6.2. Student evaluation

#### 6.2.1. Situational interest

Students’ situational interests were measured to provide information on whether the program could stimulate students’ motivation to learn. The results of students’ situational interest in the program are shown in Table 4. Regarding the individual items, the mean scores ranged from 3.67 (SD = 1.34) to 4.83 (SD = 0.38) on a five-point Likert scale ranging from 1 to 5. The dimensions that the participants rated the most were Novelty and Exploration Intention, followed by Total Interest, Instant Enjoyment, and Attention Demand. The lowest score from the participants was on the dimension Challenge. The response of this dimension also had the widest difference (SD = 1.34). This was the only participant who rated the participant’s sentence as “disagree” and “very disagree.”

**Table 4:** Summary of the PESIS.

Dimensions	Items	Strongly Disagree	Disagree	Neutral	Agree	Strongly Agree	Mean (SD)
		*N* (%)	*N* (%)	*N* (%)	*N* (%)	*N* (%)	
Novelty	Q9	0 (0)	0 (0)	0 (0)	3 (16.7)	15 (83.3)	4.83 (0.38)
	Q13	0 (0)	0 (0)	0 (0)	3 (16.7)	15 (83.3)	
	Q16	0 (0)	0 (0)	0 (0)	3 (16.7)	15 (83.3)	
	Q17	0 (0)	0 (0)	0 (0)	3 (16.7)	15 (83.3)	
Challenge	Q2	1 (5.6)	2 (11.1)	1 (5.6)	7 (38.9)	7 (38.9)	3.67 (1.34)
	Q3	2 (11.1)	3 (16.7)	2 (11.1)	6 (33.3)	5 (27.8)	
	Q20	3 (16.7)	2 (11.1)	2 (11.1)	0 (0)	11 (61.1)	
	Q3	3 (16.7)	2 (11.1)	3 (16.7)	4 (22.2)	6 (33.3)	
Exploration Intention	Q8	0 (0)	0 (0)	0 (0)	3 (16.7)	15 (83.3)	4.83 (0.38)
	Q10	0 (0)	0 (0)	0 (0)	3 (16.7)	15 (83.3)	
	Q15	0 (0)	0 (0)	0 (0)	3 (16.7)	15 (83.3)	
	Q24	0 (0)	0 (0)	0 (0)	3 (16.7)	15 (83.3)	
Instant Enjoyment	Q1	0 (0)	0 (0)	0 (0)	7 (38.9)	11 (61.1)	4.65 (0.44)
	Q12	0 (0)	0 (0)	0 (0)	4 (22.2)	14 (77.8)	
	Q14	0 (0)	0 (0)	2 (11.1)	6 (33.3)	10 (55.6)	
	Q22	0 (0)	0 (0)	0 (0)	4 (22.2)	14 (77.8)	
Attention Demand	Q4	0 (0)	0 (0)	2 (11.1)	8 (44.4)	8 (44.4)	4.53 (0.47)
	Q6	0 (0)	0 (0)	3 (16.7)	8 (44.4)	7 (38.9)	
	Q18	0 (0)	0 (0)	0 (0)	4 (22.2)	14 (77.8)	
	Q19	0 (0)	0 (0)	0 (0)	4 (22.2)	14 (77.8)	
Total Interest	Q5	0 (0)	0 (0)	0 (0)	5 (27.8)	13 (72.2)	4.75 (0.43)
	Q7	0 (0)	0 (0)	0 (0)	5 (27.8)	13 (72.2)	
	Q11	0 (0)	0 (0)	0 (0)	4 (22.2)	14 (77.8)	
	Q21	0 (0)	0 (0)	0 (0)	4 (22.2)	14 (77.8)	

#### 6.2.2. Physical fitness

Student physical fitness test results were collected and analyzed before and after the 12-week PE lessons to assess the actual efficacy of the program. Table 5 shows the results of paired sample test (parametric index) and Wilcoxon signed rank test (non-parametric index). It can be seen that after 12 weeks of PE lessons incorporated with the functional training program, significant changes (*p* < 0.01) were achieved in all physical fitness tests (BMI, VC, sit and reach, timed sit-ups, 50 m sprint, and timed rope-skipping).

**Table 5:** The effects of the functional training curriculums on measured outcomes.

Variable	Pre-test	Post-test	*N*	*Z*	*t*	*p-Value*	Effect Size		95%CI
	Mean ± SD	Mean ± SD					*d*	*r*	
BMI (kg/m^2^) ※	15.99 ± 3.30	16.49 ± 3.08	18	2.75	/	0.006^⁎^	/	0.16	(−0.77, 1.08)
VC (ml) ※	1559.17 ± 248.35	1705.83 ± 227.39	18	3.72	/	0.000^⁎^	/	0.62	(−0.33, 1.56)
Sit and reach (cm) ※	10.66 ± 5.40	12.78 ± 5.03	18	3.62	/	0.000^⁎^	/	0.41	(−0.53, 1.34)
Timed sit-ups (counts) ※	36.0 ± 6.96	41.5 ± 4.67	18	3.73	/	0.000^⁎^	/	0.93	(−0.04, 1.90)
50 m sprint (seconds)	10.71 ± 0.66	10.00 ± 0.60	18	/	−11.25	0.000^⁎^	−1.13	/	(−2.12, −0.13)
Timed rope skipping (counts)	141.44 ± 11.56	159.89 ± 10.00	18	/	11.05	0.000^⁎^	1.71	/	(0.63, 2.79)

※Non-parametric. ^⁎^Significant at *p* ≤ 0.05.

## 7. Discussion

To fill the gap in the application of functional training in primary school PE, this study designed and developed a 12-week functional training program, that could be incorporated into PE lessons, to improve students’ physical fitness. The design and development were guided by the ADDIE model. The applicability and acceptability of the program were evidenced by both the positive feedback from experts and students and the significant changes in students’ physical fitness after implementation.

When experts gave feedback on the program, most of them were positive about the program. They used words that generally express positive attitudes and express approval of the program, such as ‘全面’ (comprehensive), ‘可操作性’ (operability), and ‘可行性’ (feasibility).

Other than feedback from experts, it was also important to get feedback from students, in terms of their interest in the program. Interest is both a psychological state of attention and affection toward a particular object or topic and an enduring predisposition to reengage over time (75). It had long been recognized as a motivation factor that guides children to learn (76, 77) and a meta-analysis had reported that situational interest was a primary motivator for students to engage in PE (78). Most PE teachers focused on controlling the characteristics of situational interest to stimulate students’ situational interest, which had been proven to be positively correlated with learning engagement, to influence learning engagement (79, 80). In this study, the feedback on the students’ situational interest showed that the novelty and exploratory dimensions scored the highest. Novelty meant that the program had more novelty, and could attract participants’ attention (81). Exploratory intention indicated that participants were willing to explore a certain way of the program, which was a prerequisite for maintaining interest. This finding was in line with a study about the process of interaction between the learner and the learning content (78). The authors pointed out that learners felt the novelty and challenge of the learning content, that learners’ exploration intentions were awakened, and at the same time, they could experience a sense of enjoyment, which could stimulate their situational interest. Besides novelty and exploration, the other dimensions, such as attention demand, instant enjoyment, and total interest also had scores that were larger than 4, which meant that the program led to a high level of situational interest. As Zhou et al. (82) pointed out, functional training exercises were not only easily adapted to individual fitness levels, which could lead to a greater sense of mastery and enjoyment, but they were also fun, novel, and challenging due to the use of a variety of small, portable equipment and rapid changes in activity pace.

Conversely, the challenge dimension had the lowest score. However, it is possible that this dimension had little influence on the program as in a follow-up study to Chen and Darst (83), a path analysis had revealed that (a) instant enjoyment and exploration highly contributed positively to situational interest, (b) novelty and attention demand partially contributed positively, and (c) physical challenge contributed little (84). In this study, the participants might have been confident that they could complete the program, but it did not influence their interest in it.

As for the physical fitness performance, the results of the physical fitness test indexes showed that the baseline scores were significantly different from the post-test scores. All students had a positive growth rate and improved their physical fitness performance after the 12-week functional training program. Participants’ BMI, a sensible indication of children’s overall adiposity (85), increased dramatically but stayed within normal weight ranges (53), possibly due to increased muscle mass (86). Participants’ flexibility (sit and reach), muscular strength and endurance (timed sit-ups and timed rope-skipping), and speed (50 m sprint) all improved. It appeared that the proposed design and arrangement of functional training incorporated into PE lessons were effective in improving the students’ physical fitness. The results of this study were consistent with a systematic review that reported that functional training could improve speed, muscle strength, muscular endurance, and other physical variables (87).

This study is not without some limitations. The goodness of movement completion is influenced to some extent by the cognitive level of students in the process from movement understanding to movement execution. In addition, in the selection of movements in the program, some simple and easy movements were chosen, which to some extent reduced the intensity of the exercises (69). Finally, this study mainly focused on the control of PE classes and did not control for extracurricular activities, which may have some influence on the results of the study.

## 8. Conclusion

Children, whose health set the stage for adult health, are vital to the nation’s present and future (88). Schools, as the main place for children to improve their physical fitness (78), urgently need to conduct an in-depth study of the PE lessons to find solutions to improve the quality of the PE curriculum to improve students’ physical fitness effectively. This study was aimed at the physical fitness part of the HPECM. Under the scientific guidance of the ADDIE model, the design results of this research had not only been recognized by experts and students but also played a positive role in improving students’ physical performance. Subsequent research endeavors will implement this program in different grades of PE lessons in primary schools, conduct comprehensive teaching experiments, hope to address potential limitations, enhance the adaptability of the program further, obtain reliable and valid results, and open a window for more research on improving PE curriculum quality.

## Data availability statement

The raw data supporting the conclusions of this article will be made available by the authors, without undue reservation.

## Ethics statement

The studies involving humans were approved by The University Malaya Research Ethics Committee (Approval number: UM.TNC2/UMREC–667). The studies were conducted in accordance with the local legislation and institutional requirements. Written informed consent for participation in this study was provided by the participants’ legal guardians/next of kin.

## Author contributions

HL and JC: conceptualization and methodology. HL: data curation, software, and writing – original draft. JC: supervision and writing – review and editing. All authors contributed to the article and approved the submitted version.
